# Letter to the Editor Concerning “Risk Assessment for Toluene Diisocyanate and Respiratory Disease Human Studies”

**DOI:** 10.1016/j.shaw.2022.01.003

**Published:** 2022-02-04

**Authors:** Anne H. Chappelle

**Affiliations:** International Isocyanate Institute, Inc., Mountain Lakes, NJ, USA

To the Editor,

The above-mentioned article was recently published in Safety and Health at Work [1], with the aim of describing a model to address of healthy worker survivor effect (HWSE) bias. This is a difficult task, as many critical occupational parameters are either not available, or have drastically changed to the point that certain data should be excluded because it no longer represents modern workplaces. In order to fully validate this model and apply it to more recent data, it becomes necessary to request author clarification for a number of key points related to study design, data transparency, and overall assumptions. If left unaddressed, it is impossible to standardize the methodology and apply to other substances, or to repeat with accuracy. Summarized below are selected examples to demonstrate ambiguity and concerns, with further detail provided in four accompanying Attachments.

**Healthy worker effect (HWE)** – While we agree that HWE should be controlled [2], the underlying assumption that HWE would be dependent on exposure level is not supported by the data. As detailed in Attachment 1 Adams [3], one of the only cohorts to provide sufficient within-study information for analysis, describes a high percentage of participants that left for medical reasons during their first year of employment which did not correlate with TDI exposure levels during that year. Consideration of HWE in workers sensitized to diisocyanates is a fundamental component of epidemiology studies because removal is a long-established and effective way to improve prognosis [[5], [6], [7]]. Assuming workers that leave have increased mortality is without supporting data and further confounds analysis.

**Modeling dose–response** – Not all assumptions made by [1] are supported in literature (e.g., [8]). For example, it is surprising that “*a linear exposure-response for asthma/sensitization incidence and TDI concentration*” had to be assumed to enable the analysis (“*without linearity, different studies could not be meta-summarized*”) (2.2 – p. 175), whereas both in [1] Figure 1 as well as another similar analysis [9] clearly demonstrate such dose–response to be absent at low exposure. A lack of dose–response likely results from a combination of factors beyond HWE (ex. peak exposure, dermal contact, chemical co-exposures [[10], [11], [12]], or even the use of the unspecific average exposure), none of which were addressed. Attachment 2, which is a cross-check of data presented in [1] Table S2 against original publications, repeatedly reveals (a) inconsistencies, and (b) potential bias from assumptions made regarding exposure concentrations. Because the model clearly has a non-significant slope parameter ([1] SOM2 - confidence interval [-5.3 + 5.7]), and taking into account comments made in the next paragraph, conclusions lack adequate justification, which invalidates the findings.

**Use of exposure response per ppb (XR)** – In absence of a dose–response, or even considering that incidence may relate to exposure with an exponent < 1 (log-based logistic regressions reduce to power functions at low exposure) [13], the ratio of incidence rate (IR) and exposure (XR = IR/X) is a function with a negative exponent in X. This is interpreted to mean that XR has an infinite value and slope at X = 0, hence representing it by an exponential function ([1] SOM2) artificially creates intercepts. Additional clarity and justification for this analysis is necessary, as there is not convincing evidence that XR and HWE are linked.

**Lung function** – The general statement that TDI “*induces … diminishing pulmonary function …*” (1 – p.174) and the assumption that this effect is cumulative (2.3 – p. 176) are not supported by studies in [1] Table S3. Because of a lack of transparency in the criteria for study inclusion, it is concerning that studies that provide contradicting assumptions are excluded [ex. 14,15]. Attachment 3, which cross-checks [1] Table S3 against original publications, demonstrates (a) excess loss of lung function may have been overestimated, (b) presence of selection bias (e.g., subgroups of studies not considered), and (c) additional unsupported assumptions made about exposure concentrations. For example, attributing all-cause FEV1-related mortality data (2.3 – p. 176) solely to TDI is questionable. Without additional explanation, there is significant doubt about the validity of conclusions reached (3.2 – p. 178).

**Carcinogenicity and non-malignant respiratory diseases (NMRD)** – The statement “*TDI … has also been associated with increased lung cancer*” (p. 174) refers to three cited studies [[16], [17], [18]]. Attachment 4 provides specific examples how the attribution has been distorted in [1] and in fact do not support associating TDI with lung cancer nor “*Assuming a) excess NMRD deaths were* ***attributable*** *to TDI-related exposures …*” (2.4 – p. 177 – emphasis added) and erodes the basis for the [1] analysis. Assuming that non-significant associations between NMRD and cancer mortality are solely attributable to a HWE (3.3 – p.179) that was in itself assumed to be applicable to cancer mortality “*because … (it) was observed for asthma*” (2.4 – p. 176), implicitly bears additional assumptions (a) that mortality of participants that left studies early would be causally related to their short exposure, and (b) that such exposure would continue to have a life-time post-exposure impact. With three studies showing no significant relationship between TDI exposure and either lung cancer or NMRD mortality, the claim that 5% of the female workforce would die of lung cancer because of TDI exposure (4.1 – p. 181), is not consistent with biological plausibility and warrants reevaluation of model assumptions.

**Conclusion** – The abundance of questions and concerns raised and the introduction of unverified but fundamental assumptions warrant significant clarification in order to validate [1] with new data, and/or accurately apply to other respiratory sensitizers.

## Conflicts of interest

The author is employed by the International Isocyanate Institute, Inc. The Institute is funded by producers of TDI and MDI. The opinions expressed herein are those of the author, not of the Institute or its member companies.

## References

[1] Park R.M. (2021). Risk assessment for toluene diisocyanate and respiratory disease human studies. Saf Health Work.

[2] Le Moual N., Kauffmann F., Eisen E.A., Kennedy S.A. (2008). The healthy worker effect in asthma. Work may cause asthma, but asthma may also influence work. Am J Respir Crit Care Med.

[3] Adams W.G. (1975). Long-term effects on the health of men engaged in the manufacture of tolylene di-isocyanate. Br J Ind Med.

[5] Bernstein D.I. (1992). Occupational asthma. Clin Allergy.

[6] Ott M.G., Diller W.F., Jolly A.T. (2003). Respiratory effects of toluene diisocyanate in the workplace: a discussion of exposure-response relationships. Crit Rev Toxicol.

[7] Uppal N., Bhinder S., Ribeiro M., Chaudhry S., Stanbrook M.B., Liss G., Tarlo S.M. (2016). Prognosis of diisocyanate-induced occupational asthma: a systematic review. Am J Respir Crit Care Med.

[8] Pauluhn J., Whalan J.E. (2021). Human risk assessment of inhaled irritants: role of sensory stimulations from spatially separated nociceptors. Toxicology.

[9] Daniels R.D. (2018). Occupational asthma risk from exposures to toluene diisocyanate: a review and risk assessment. Am J Ind Med.

[10] Belin L., Wass U., Audunsson G., Mathiasson L. (1983). Amines: possible causative agents in the d development of bronchial hyperreactivity in workers manufacturing polyurethanes from isocyanates. Br J Ind Med.

[11] Clark R.L., Bugler J., McDermott M., Hill I.D., Allport D.C., Chamberlain J.D. (1998). An epidemiology study of lung function changes of toluene diisocyanate foam workers in the United Kingdom. Int Arch Occup Environ Health.

[12] Plehiers P.M., Chappelle A.H., Spence M.W. (2020). Practical learnings from an epidemiology study on TDI-related occupational asthma: Part I—cumulative exposure is not a good indicator of risk. Toxicol Ind Health.

[13] Collins J.J., Anteau S., Conner P.R., Cassidy L.D., Doney B., Wang M.L., Kurth L., Carson M., Molenaar D., Redlich C.A., Storey E. (2017). Incidence of occupational asthma and exposure to toluene diisocyanate in the United States toluene diisocyanate production industry. J Occup Environ Med.

[14] Wang M.L., Storey E., Cassidy L.D., Doney B., Conner P.R., Collins J.J., Carson M., Molenaar D. (2021). Longitudinal and cross-sectional analyses of lung function in toluene diisocyanate production workers. J Occup Environ Med.

[15] Bodner K.M., Burns C.J., Randolph N.M., Salazar E.J. (2001). A longitudinal study of respiratory health of toluene diisocyanate production workers. J Occup Environ Med.

[16] Mikoczy Z., Welinder H., Tinnerberg H., Hagmar L. (2004). Cancer incidence and mortality of isocyanate exposed workers from the Swedish polyurethane foam industry: updated findings 1959-98. Occup Environ Med.

[17] Sorahan T., Nichols L. (2002). Mortality and cancer morbidity of production workers in the UK flexible polyurethane foam industry: updated findings, 1958-98. Occup Environ Med.

[18] Pinkerton L.E., Yiin J.H., Daniels R.D., Fent K.W. (2016). Mortality among workers exposed to toluene diisocyanate in the US polyurethane foam industry: update and exposure-response analyses. Am J Ind Med.

