# Supplementary material for: Letter to the Editor Concerning “Risk Assessment for Toluene Diisocyanate and Respiratory Disease Human Studies”
Source: Saf Health Work. 2022 Feb 4;13(1):129–30. doi: 10.1016/j.shaw.2022.01.003 (PMC9347006; doi:10.1016/j.shaw.2022.01.003)
Supplement: Multimedia component 2 [file mmc2.docx]

**Attachment 2**

**Comments on data in Supplementary Online Materials 2 (SOM 2) of Park [1]**

Table S2-1 summarizes estimates of TDI-induced asthma or symptoms in populations exposed to TDI. There are more publications available than reviewed by [1], but selection criteria were not reported.

Table S2-1 – Overview of studies listed in Table S2 (SOM2) of [1]. References as in [1]. Items **highlighted in red** cannot be traced back to the original publications.

| Reference | Product | # Cases | # Participants | Duration  [years] | Person-Years  [ps-year] | TDI Concentration  [ppb] | Incidence Rate  [1/(100 ps-year)] |
| --- | --- | --- | --- | --- | --- | --- | --- |
| Bruckner (1968) [2] | TDI | 5 | 26 | 5 | **130** (1) | **20** (3) | **3.85** (2) |
|  | 1. This study reported exposure duration of 0.25 to 11 years. The number of person-years of exposure cannot be determined. 2. All cases acquired sensitization prior to 1963. The study ran for a longer period of time, hence incidence rate is likely underestimated. 3. Exposure at the time period prior to 1963 was reported as [Range (median) in ppb]: 0-240 (33), 0-190 (35), or 100-500 (77), depending upon the exposure group. Exposure concentrations were much higher than the 20 ppb used. | | | | | | |
| Adams (1975) [3] | TDI | 82 (2) | **268** (2) (4) (5) | **9** (2) | **1593** | **73** (1) (3) | **5.15** |
|  |  | 7 | **268** | **9** | **246** | **20** | **2.85** |
|  |  | 11 | **268** | **9** | **117** | **10** | **9.40** |
|  |  | 24 | **268** | **9** | **308** | **5** | **7.79** |
|  | 1. This study did not report exposure concentrations, but merely indicated a percentage of samples exceeding 20 ppb, together that exposure in 1962-1964 was “usually between 50-100 ppb”. The values reported by Park are assumptions. The personnel numbers reported by Adams are an annual census. 2. The 82 cases belonged to the group of (256+118) workers that was employed for the full 9 years of the study; the 7 cases belonged to the group (initially 52 workers) that was employed for 8 years; the 11 cases were in the group (initially 34 workers) with 7 years of study participation; and the 24 cases were hired later and had 6 or less years of study participation (105 workers). Hence, the number of participants is obviously incorrect, as is consequently the number of person-years and the incidence rate. 3. The exposure concentrations cannot be associated with the groups the way Park does, because calendar year and participation year do not match in Table 1 of Adams. 4. A brief analysis of the data presented in Table 1 of Adams shows that the average incidence rate for the study should be approx. 5.4%. In the first year of employment (Y1), it is approx. 15%, in the two subsequent years (Y2-Y3) ca. 3%, and 1.0-1.5% in the subsequent years. These percentages are more or less independent of the date of hire. Consequently, since exposure concentrations decreased with proceeding calendar time, there is no obvious relationship between exposure and participants leaving for medical reasons. See Supplement 1. 5. Adams reported all leavers incl. the reason why. The brief analysis mentioned sub (4) therefore took into account all HWE-related effects. The results seem to suggest that there exists a group highly sensitive people but does not provide evidence of a dose-dependent HWE. | | | | | | |
| Porter (1975) [4] | TDI | 11 | 200 | 3.5 | 700 (1) (2) | **57** (3) (4) | 1.57 (5) |
|  |  | 16 | 200 | 11 | 2000 | **40** | 0.80 |
|  |  | 3 | 200 | 5 | 1000 | **14** | 0.30 |
|  | 1. Neither the number of individual exposure years, nor the overall person-years can be determined based on the published information. Porter reports a turnover of approx. 100 persons. 2. It is not correct to link the year of diagnosis with the exposure concentration in that year, since many participants had long employment histories. The subdivision in three groups was made by Park, not reported by Porter, and seems not recommendable, since average exposure concentrations remained virtually the same for almost 15 years. 3. Porter attributes the reduction in cases over time to (a) a potential hardening of the workforce (could be a HWE) and (b) to reduction of exposure excursions. The latter was unaccounted for in Park’s analysis. 4. The exposure numbers are an interpolation by Park, not published by Porter. 5. The average overall incidence rate in this study would be approx. 1% at an average exposure of ca. 50 ppb. | | | | | | |
| Franzinelli (1978) [5] | PU | 16 (1) | **66** (1) | 2.75 | **181.5** | **19** (3) | **8.82** (2) |
|  | 1. This study reported the number of cases in a quite confusing fashion. There were 2 cases of asthma identified in the group of 66 “currently exposed” workers. There were however 14 more cases identified in the group of 81 “leavers” (no reason given), for which no duration of exposure was recorded. Chronic bronchitis was observed in 14 additional workers of the “currently exposed” group. 2. Hence, the reported asthma incidence rate is high and should be corrected to 16/(66+81) = 3.94%. 3. Franzinelli did not report exposure concentrations, only frequencies within certain ranges. Exposure ranged between 0-500 ppb. Assuming a log-normal distribution of exposure concentrations, the frequencies would support an average exposure concentration of 30-32 ppb. The 19 ppb mentioned by Park cannot be traced to the original publication. | | | | | | |
| Weill (1981) [6] (1) (2) | TDI | 12 | 218 (5) | 5.5 | 1199 | 3.5 | 1.00 (3) (4) |
|  | 1. This study forms the basis of the Diem (1982) publication, which was excluded in the Park article. 2. The study is interesting because it was started with a naïve population (plant start-up). 3. There was a higher incidence of asthma cases in the low cumulative exposure group (1.2%) compared to the high cumulative exposure group (0.9%) (Plehiers et al. Toxicol Ind Health 2020;36(11):876-884 – Table 1). This does not support a cumulative effect; but is rather indicative of the existence of a group of highly sensitive individuals (see Adams). 4. The conclusion of Diem (1982) that there is a link between cumulative exposure and lung function is an opposite trend vis-à-vis asthma incidence. 5. The study would in principle allow to split up the population in 3 different exposure groups. | | | | | | |
| Belin (1983) [7] | PU | 3 | 48 | 6 | **288** (1) | 1.5 | **1.04** (1) (2) |
|  | 1. The incidence rate mentioned is based on the sole statement that 3 out of 48 employees were transferred in the course of a 6-year period because of effects. No duration of exposure prior to symptoms was reported. No diagnosis method was reported either. 2. The incidence rate should be considered low for that reason. | | | | | | |
| Wang (1988) [8] | OTHER | 14 | 34 | 0.77 | 26.2 | **27.5** (2) (3) | 53.5 (1) |
|  | 1. This was a cross-sectional study and only prevalence was reported. This was transformed into an incidence rate using the published average 9.2 months of average employment (extreme turnover). Four workers were excluded by Wang because of a smoking history. 2. A high probability of dermal exposure can be suspected (application of adhesives in an otherwise poorly set-up workplace). With potentially high temperatures during the stitching process, exposure to other chemicals cannot be excluded. 3. Wang reported atmosphere concentrations in three zones. The differentiation could have been reported. In the zone with exposure of 12 +/- 2 ppb, 0/13 workers showed asthma symptoms. In the zone with 21 +/- 6 ppb exposure, 3/8 showed symptoms, and in the zone with 47 +/- 54 ppb exposure, 11/13 workers had symptoms. This shows a clear dose-response at elevated concentrations. | | | | | | |
| Huang (1991) [9] | OTHER | 4 | 15 | 7.7 | 115.5 | 112 (1) (2) | 3.46 |
|  | 1. Study on spray painters. Exposure was very high, and included other isocyanates as well. Use of protective equipment cannot be judged. 2. A low exposure group (with zero cases) was reported as well but is not included here. | | | | | | |
| Daftarian (2000) [10] (2) | PU | 20 | 114 | 13 | **3800** (1) | 0.23 | **0.53** (1) |
|  | 1. The person-years don’t match with the participants and study duration. Consequently, incidence is also incorrect, and should be corrected upwards to 1.36%. Daniels (2018) reported 1482 person-years, which would seem to be correct. This value was used for the corrected incidence rate. 2. Of note: this study was considered an outlier and rejected by Daniels. | | | | | | |
| Mean (1) | -- | 13.8 | 182 |  |  | 20.6 | 4.70 |
|  | 1. One should not calculate the mean of cases, exposure and incidence rates by simple arithmetic averaging. | | | | | | |

Taking into account the comments made in this Table S2-1, a revised graph similar to the one shown as Figure 1 by [1] is given in Figure S2-1. At higher TDI concentrations, there seems to be a dose-response. At TDI concentrations below ca. 3 ppb (log(X)=0.5), the dose-response seems to be absent. The latter is consistent with data presented by [11].


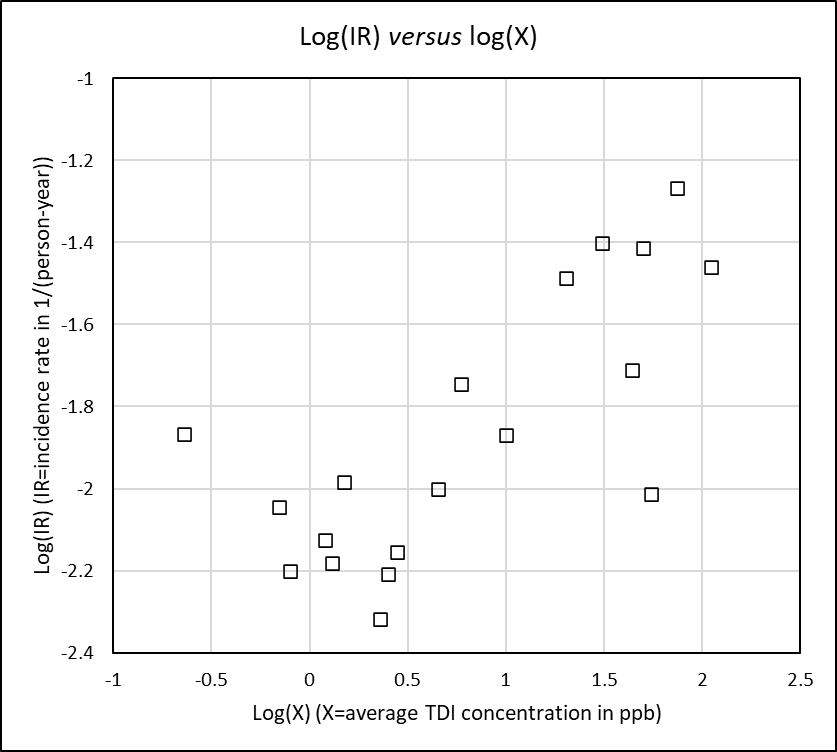


Figure S2-1 – Incidence rate plotted against average TDI exposure concentration for the studies reported in SOM2 of Park (2021) with corrections as indicated in Table 1.

References:

[1] Park RM. Risk assessment for toluene diisocyanate and respiratory disease human studies. Safety and Health at Work. 2021;12(2):174-83. doi: 10.1016/j.shaw.2020.12.002.

[2] Bruckner HC, Avery SB, Stetson DM, Dodson VN, Ronayne JJ. Clinical and immunologic appraisal of workers exposed to diisocyanates. Archives of Environmental Health. 1968;16:619-25.

[3] Adams WGF. Long-term effects on the health of men engaged in the manufacture of tolylene di-isocyanate. British Journal of Industrial Medicine. 1975;32:72-8.

[4] Porter CV, Higgins RL, Scheel LD. A retrospective study of clinical, physiologic and immunologic changes in workers exposed to toluene diisocyanate. American Industrial Hygiene Association Journal. 1975;36(3):159-68.

[5] Franzinelli A, Mariotti F, Innocenti A. Respiratory pathology of isocyanates in a refrigerator factory. La Medicina del Lavoro. 1978;69:163-70.

[6] Weill H, Butcher B, Dharmarajan V, Glindmeyer H, Jones R, Carr J, et al. Respiratory and immunologic evaluation of isocyanate exposure in a new manufacturing plant. NIOSH Technical Report Publication No 81-125: Tulane University; 1981. p. 152P,40Ref,29Tab,22Fig.

[7] Belin L, Wass U, Audunsson G, Mathiasson L. Amines: possible causative agents in the development of bronchial hyperreactivity in workers manufacturing polyurethanes from isocyanates. British Journal of Industrial Medicine. 1983;40:251-7.

[8] Wang JD, Huang PH, Lin JM, Su SY, Wu MC. Occupational asthma due to toluene diisocyanate among Velcro-like tape manufacturers. American Journal of Industrial Medicine. 1988;14:73-8.

[9] Huang J, Wang XP, Ueda A, Aoyama K, Chen BM, Matsushita T. Allergologic evaluation for workers exposed to toluene diisocyanate. Industrial Health. 1991;29:85-92.

[10] Daftarian HS, Roegner KC, Reh CM. Health Hazard Evaluation Report: Woodbridge Corporation, Brodhead, Wisconsin. HETA 98-0011-2801: National Institute for Occupational Safety and Health; NIOSH; 2000. p. 63P,76Ref,20Tab.

[11] Daniels RD. Occupational asthma risk from exposures to toluene diisocyanate: a review and risk assessment. American Journal of Industrial Medicine. 2018:[Epub ahead of print]. doi: 10.1002/ajim.22815.
