# Supplementary material for: Letter to the Editor Concerning “Risk Assessment for Toluene Diisocyanate and Respiratory Disease Human Studies”
Source: Saf Health Work. 2022 Feb 4;13(1):129–30. doi: 10.1016/j.shaw.2022.01.003 (PMC9347006; doi:10.1016/j.shaw.2022.01.003)
Supplement: Multimedia component 3 [file mmc3.docx]

**Attachment 3**

**Comments on data in Supplementary Online Materials 3 (SOM3)**

Table S3-1 summarizes findings of pulmonary function tests in populations exposed to TDI. There are more publications available than reviewed by [1], but selection criteria were not reported. The designation “# Experiments” stems from the SOM of [1], whereby the number often only represents part of the study populations.

Table S3-1 - Overview of studies listed in Table S3 (SOM2) of Park [1]. References as in Park [1]. Items **highlighted in red** cannot be traced back to the original publications.

| Reference | # Experiments | Cumulative Exposure  [ppb-years] | TDI Concentration  [ppb] | Type | Outcome | Outcome units | Stipulated Value |
| --- | --- | --- | --- | --- | --- | --- | --- |
| Jang (2000) [2] (3) (4) (5) | 44 | 21.1 | 2.5 | FEV1 | 81.3 (2) | % of predicted | **106 / 85.6** (1) |
|  | 44 | 21.1 | 2.5 | FVC | 84.3 | % of predicted | **106 /88.4** |
|  | 1. Jang reported reference values for the control group (FEV1 85.6% of predicted; FVC 88.4% of predicted). There is no clear purpose to stipulate a value; the generation and use of the stipulated value in this case artificially increases the effect. 2. Jang results represent a single measurement in a cross-sectional study. There is no report of significance of the difference between TDI exposed participants and the control group; there is no sign of obstructive lung function (FEV1/FVC is the same for TDI exposed and control groups). 3. Prevalence of airway hyperresponsiveness was not significantly different for TDI exposed compared with control groups. 4. There is no correction applied for other confounding factors (e.g., smoking). 5. As a result, excess loss is likely overestimated in the Park approach. | | | | | | |
| Omae (1992) [3] (5) | 29 | 94.1 (4) | **5.7** (1) | FEV1 | 0.56 (3) | % loss per year | **103** |
|  | 29 | 94.1 | **5.7** | FVC | 0.19 (2) | % loss per year | **104** |
|  | 28 | 1.7 | 0.1 (1) | FEV1 | 0.38 (3) | % loss per year | **103** / **100.6** |
|  | 28 | 1.7 | 0.1 | FVC | 0.22 (2) | % loss per year | **104** / **102.2** |
|  | 1. Omae also reported a split of the high exposure group into H1 and H2, with average exposures of 8.2 and 1.7 ppb respectively. A reference group was reported as well. 2. FVC trends upward with exposure (loss becomes negative): %AAL of Ref = 0.02, L = 0.22, H2 = 0.49, H1 = -0.10. In mL/yr, from the study records (unpublished), average declines in FVC were Ref = 27+/-46, L = 37+/-39, H2 = 38+/-46, and H1 = 17+/-64. 3. % AAL FEV1 for Ref, L and H2 groups (up to 1.7 ppb) is not noticeably different: Ref = 0.34, L = 0.38, H2 = 0.28, but is significantly different for the H1 group (0.81). In mL/yr, from the study records (unpublished), average declines in FEV1 were Ref = 46+/-39, L = 47+/-32, H2 = 40+/-36, and H1 = 64+/-69. 4. Study duration was 4 years; pre-employment was 16-17 years. Not relevant for the considerations, since cumulative exposure isn’t used in the fit. 5. Given that – with the exception of the H1 group – loss was not different from the Reference group, excess loss is clearly overestimated in the Park approach. | | | | | | |
| Wegman (1982) [4] (4) | 48 | 14.4 | **3.6** (1) | slope | **4.94** (2) (3) | mL/(4 yr)/(ppm*1000) | 3.8 (L) |
|  | 1. Wegman reported three exposure groups: <2ppb, 2-3.4 ppb, and >3.5 ppb. No further details are given. The 3.6 ppb exposure concentration is an assumption and represents only one group of the Wegman study. 2. For the low exposure group, Wegman reports 4-year FEV1 decrements that are not elevated compared to normal evolution. For the medium exposure group, 4-year FEV1 decrements are only elevated for smokers. For the high exposure group, 4-year decrements are elevated for all groups. 3. The correlation parameters determined by Wegman were a 4-year decrement of 4.94 mL per ppm*1000 average exposure concentration (not cumulative), and a 4-year increment of 1.54 mL increment per month of employment, which was not considered by Park. The combination of the two parameters is necessary to properly represent the effects mentioned under (2). 4. The implication of these assumptions is that excess loss is therewith clearly overestimated in the Park approach. | | | | | | |
| Clark (1998) [5](5) | 157 (3) | 5.04 | **1.2** (4) | FEV1 | **36** (1) (2) | mL/yr | 3.8 (L) |
|  | 1. Clark [Abstract]: *… the annual declines of … FEV1 and … FVC were not related to TDI exposure and were typical of those observed in other longitudinal populations … This study does not provide evidence that there is a TDI-related decline in FEV1 and FVC in workers exposed to less than … 5.8 ppb.* This is clear from Table 5 of Clark. 2. The 36 mL/yr excess decrement relates to naïve workers. Clark reports (p. 174) …*analysis of those naives in the study for more than 4 years showed age as the only significant variable for annual changes in FEV1 and FVC. Regressions on annual change in FEV1 for shorter periods showed in-study mean daily exposure as the only significant variable. The excess decline would be limited to a period of 4 years.* 3. The 157 refers to the number of naïve study participants out of a total of 780. Of these 157, 8 belonged to the low exposure group, 30 to the handling group, and 119 to the exposed group (Clark Table 2). 4. Clark reports that 79% of participants were exposed to less than 10 ppb-hr per day (corresponding to the 1.2 ppb assumed by Park). However, much higher exposures were reported in both the handling and exposed groups that 30, respectively 119 of the naïve participants belonged to. Details about the exposure of naïve participants were not reported by Clark. 5. As a consequence, excess loss seems to be overestimated. | | | | | | |
| Clark (2003) [6] (5) | 26 |  | 0.6 (3) (4) | FEV1 | **0.00674** (1) (2) | L/yr compared to low exposure group | 3.4 (L) |
|  | 26 |  | 0.6 | FVC | **0.017** | L/yr compared to low exposure group | 4.0 (L) |
|  | 1. The slope parameters used are those for the handling group only (26 out of a total of 251 participants). Clark reports a difference in lung function for this group compared to the low exposure group. 2. Clark also reports information for the high exposure group (175 participants) for which such decrement is absent. It is unclear why the high exposure group has not been included in the Park analysis. 3. From Clark (p. 300): *Lung function measurements taken by the same investigator using the same calibrated equipment, on two occasions up to 17 years apart, showed the expected relationships with age, gender, smoking habits and weight gain. They showed no harmful relationship with TDI exposure at the levels prevailing at the time in the industry as calculated from measured exposures to TDI and occupational histories.* 4. Clark (1998) also mentions the use of adhesives and the presence of amines in the working environment of the handling group as a potential confounder. See also Belin (1983), Omae (1992). Gui et al. (2014) have observed increased respiratory symptoms in a similar group of workers. 5. As a result of these assumptions, the relationship between exposure and lung function decline is potentially distorted by not including the high exposure group. Excess loss seems to be overestimated, or not accurately represented. | | | | | | |
| Ott (2000) [7] (4) |  | 19.7 | 4.2 | FEV1 | **0.013** / **0.00013** (1) (2) | ?? /  L/(ppb-month) | **3 (??)** |
|  | 1. The slope parameter for lung function decrement as a function of cumulative exposure (blue value) was forced into the model by Ott. From Table 5 in Ott, it is obvious that the parameter is not significant (not marked as such, and the error on the estimate is of the same magnitude as the estimate). Non-significant parameters would normally be eliminated from a model. 2. Correctly, Ott reports (p. 43) … *there was no relationship between cumulative exposure to TDI and irreversible air flow obstruction as assessed by spirometry* … (p. 49) *The TDI concentration and cumulative dose were not significant predictive factors in the full model* … 3. We cannot verify how the red value was derived. The blue value was taken from the original publication. 4. It would appear that the parameter used was taken out of context. Excess loss seems to be overestimated. | | | | | | |
| Holness (1984) [8] | 95 | 16.3 | 2.5 | FEV1 | **4.5** (1) (2) | % change over working shift | 107.9 |
|  | 95 | 16.3 | 2.5 | FVC | **3.2** | % change over working shift | 108.8 |
|  | 1. Holness reported lung function changes over a work shift. This is an acute response only and is not indicative of a chronic effect. 2. We are therefore of the opinion that the Holness results should not have been used for the Park analysis. | | | | | | |
| Jones (1992) [9] | 287 (1) (3) | 23.5 | 2.5 | FEV1 | **4.3** (1) (2) (3) | %/100 ppb-month | 107.9 |
|  | 287 | 23.5 | 2.5 | FVC | **4.4** | %/100 ppb-month | 107.1 |
|  | 1. Jones reports parameters per smoking group in Table 7. For Ex- and Never-smokers, the effect is small and the parameter is not significant. The declines used refer to the group of current smokers only, but has been applied to the entire study population of 287. 2. In Table 8, Jones reports a non-significant (p=0.8) but positive (=increase) effect on lung function after controlling for smoking and age. 3. Park has selected the only group with significant effect (but also a significant confounding factor – “current smokers”) into his analysis. This is bound to have introduced bias. Single group selection from other data sets has been noted already. | | | | | | |
| Wang (2017) [10] (1) | 178 |  | 0.7 | FEV1 | (2) |  |  |
|  | 1. By way of example of studies not included. 2. Parameter estimates in a multi-variable model show FEV1 decline to be not significantly related to cumulative TDI exposure (Tables 5 – p. S33 – p=0.50 for the entire group of 178 participants; and 6 – p. S33 – p=0.15 when only considering the 118 white male participants). | | | | | | |

REFERENCES:

[1] Park RM. Risk assessment for toluene diisocyanate and respiratory disease human studies. Safety and Health at Work. 2021;12(2):174-83. doi: 10.1016/j.shaw.2020.12.002.

[2] Jang AS, Choi IS, Koh Y, Moon JD, Lee KJ. Increase in airway hyperresponsiveness among workers exposed to methylene diphenyldiisocyanate compared to workers exposed to toluene diisocyanate at a petrochemical plant in Korea. American Journal of Industrial Medicine. 2000;37(6):663-7.

[3] Omae K, Higashi T, Nakadate T, Tsugane S, Nakaza M, Sakurai H. Four-year follow-up of effects of toluene diisocyanate exposure on the respiratory system in polyurethane foam manufacturing workers. II. Four-year changes in the effects on the respiratory system. International Archives of Occupational and Environmental Health. 1992;63:565-9.

[4] Wegman DH, Musk AW, Main DM, Pagnotto LD. Accelerated loss of FEV-1 in polyurethane production workers: a four-year prospective study. American Journal of Industrial Medicine. 1982;3:209-15.

[5] Clark RL, Bugler J, McDermott M, Hill ID, Allport DC, Chamberlain JD. An epidemiology study of lung function changes of toluene diisocyanate foam workers in the United Kingdom. International Archives of Occupational and Environmental Health. 1998;71:169-79.

[6] Clark RL, Bugler J, Paddle GM, Chamberlain JD, Allport DC. A 17-year epidemiological study on changes in lung function in toluene diisocyanate foam workers. International Archives of Occupational and Environmental Health. 2003;76:295-301.

[7] Ott MG, Klees JE, Poche SL. Respiratory health surveillance in a toluene di-isocyanate production unit, 1967-97: clinical observations and lung function analyses. Occupational and Environmental Medicine. 2000;57(1):43-52.

[8] Holness DL, Broder I, Corey PN, Booth N, Mozzon D, Nazar MA, et al. Respiratory variables and exposure-effect relationships in isocyanate-exposed workers. Journal of Occupational Medicine. 1984;26(6):449-55. doi: 10.1097/00043764-198406000-00018.

[9] Jones RN, Rando RJ, Glindmeyer HW, Foster TA, Hughes JM, 0'Neil CE, et al. Abnormal lung function in polyurethane foam producers: Weak relationship to measured TDI exposures. The American Review of Respiratory Disease. 1992;146:871-77.

[10] Wang ML, Storey E, Cassidy LD, Doney B, Conner PR, Collins JJ, et al. Longitudinal and cross-sectional analyses of lung function in toluene diisocyanate production workers. Journal of Occupational and Environmental Medicine. 2017;59(Suppl.12):S28-35. doi: 10.1097/jom.0000000000001124.
