# Supplementary material for: Letter to the Editor Concerning “Risk Assessment for Toluene Diisocyanate and Respiratory Disease Human Studies”
Source: Saf Health Work. 2022 Feb 4;13(1):129–30. doi: 10.1016/j.shaw.2022.01.003 (PMC9347006; doi:10.1016/j.shaw.2022.01.003)
Supplement: Multimedia component 4 [file mmc4.docx]

**Attachment 4**

**Quotations from TDI cancer studies**

(References given in main paper)

Quotations (*italicized*) from studies cited in Park [1] strongly put into question an approach that attributes all observed morbidities to TDI. Statements quoted from Park do not always seem to be very precise when compared with the original statements. In particular, association with TDI is being inferred without being supported by the referenced studies.

Sorahan and Nichols (2002) [2]

(p. 751) *Compared to the general population of England and Wales* … *There were no significantly increased cause specific SMRs … with some period of isocyanate exposed employment.*

(p. 757) *All female lung cancer cases occurred in women without any period of isocyanate exposed employment, …*

(p. 758 – NMRD in female workers) *risks for this disease grouping were not found to be associated with duration of isocyanate exposed employment.*

(p. 751) *The study has been unable to link isocyanate exposed employment either with risks of lung cancer or with risks of non-malignant diseases of the respiratory system. The increased SMR for female lung cancer is most likely caused by factors unrelated to the industry under study.*

Mikoczy et al. (2004) [3]

(p. 432) *Results support the findings from two other cohort studies of an increased lung cancer risk among female workers in the polyurethane foam manufacturing industry. … However, the study was not able to link isocyanate exposed employment with lung cancer risk.*

(p. 432) … *the lung cancer incidence was enhanced in women. Women with ‘‘apparent exposure’’ to TDI or MDI did not, however, have a higher lung cancer incidence than those with ‘‘no or low exposure’’.*

(p. 436) *Thus, there is no convincing evidence that exposure to isocyanates in the polyurethane foam manufacturing industry has resulted in increased mortality in obstructive lung diseases.*

Pinkerton et al. (2016) [4]

(p. 1) *Lung cancer mortality was increased but was not associated with exposure duration or cumulative TDI exposure.*

(p. 1) *In post hoc analyses, lung cancer mortality was associated with employment duration in finishing jobs, but not in finishing jobs involving cutting polyurethane foam.*

(p. 1) *Limitations include the lack of smoking data, uncertainty in the exposure estimates, and exposure estimates that reflected inhalational exposure only.*

(p. 8) *Mortality …, COPD, … was highest in the lowest exposure duration category, and a significant negative trend in mortality from COPD with exposure duration was observed. Mortality from COPD and intentional self-harm was also highest in the lowest cumulative TDI exposure category, but a significant negative trend with cumulative TDI exposure was only observed for intentional self-harm.*

(p. 12) *Mortality from cancers of the breast, intestine, and brain, and NHL was elevated in the cohort, although not significantly, and significantly associated with exposure duration or cumulative TDI exposure estimates. Breast cancer was associated with cumulative TDI exposure in the main analysis, but not in analyses excluding short-term workers.*

(p. 12) *Breast cancer mortality and/or incidence was elevated in men, but not in women in the most recent updates of the UK and Swedish cohorts, and the findings in men were based on very few observed cases and not statistically significant [Sorahan and Nichols, 2002; Mikoczy et al., 2004].*

Park (2021) [1]

(p. 174) *Three studies show an excess of lung cancer in women workers exposed to TDI or associated intermediates and degradation products.*

(p. 176) referring to [2] as well as [4]: *Two other TDI studies* *found elevated mortality for NMRD, especially for women, compared with that typically observed in industrial populations*.

(p. 176): *Pinkerton et al. analyzed mortality in a NIOSH TDI cohort updated through 2011, which was available for this work; their analyses revealed statistically significant excesses for lung cancer and non-malignant respiratory diseases (NMRDs) compared with the general population, especially for women, but no significant positive associations with duration of, or cumulative, exposure to TDI.*

(p. 176) also referring to [4]: *A significant trend was observed for female breast cancer mortality on TDI cumulative exposure (p = 0.02) and TDI duration (p = 0.017).* [Note: significant trend with exposure, but no significant increase].

**References**

[1] Park RM. Risk assessment for toluene diisocyanate and respiratory disease human studies. Safety and Health at Work. 2021;12(2):174-83. doi: 10.1016/j.shaw.2020.12.002.

[2] Sorahan T, Nichols L. Mortality and cancer morbidity of production workers in the UK flexible polyurethane foam industry: updated findings, 1958-98. Occupational and Environmental Medicine. 2002;59(11):751-8.

[3] Mikoczy Z, Welinder H, Tinnerberg H, Hagmar L. Cancer incidence and mortality of isocyanate exposed workers from the Swedish polyurethane foam industry: updated findings 1959-98. Occupational and Environmental Medicine. 2004;61(5):432-7.

[4] Pinkerton LE, Yiin JH, Daniels RD, Fent KW. Mortality among workers exposed to toluene diisocyanate in the US polyurethane foam industry: Update and exposure-response analyses. American Journal of Industrial Medicine. 2016;59(8):630-43. doi: 10.1002/ajim.22622.
